# Hypoxia classifier for transcriptome datasets

**DOI:** 10.1186/s12859-022-04741-8

**Published:** 2022-05-31

**Authors:** Laura Puente-Santamaría, Lucia Sanchez-Gonzalez, Ricardo Ramos-Ruiz, Luis del Peso

**Affiliations:** 1 Departamento de Bioquímica, Universidad Autónoma de Madrid (UAM), 28029 Madrid, Spain; 2 Instituto de Investigaciones Biomédicas “Alberto Sols” (CSIC-UAM), 28029 Madrid, Spain; 3 Genomics Unit Cantoblanco, Fundación Parque Científico de Madrid, C/ Faraday 7, 28049 Madrid, Spain; 4 IdiPaz, Instituto de Investigación Sanitaria del Hospital Universitario La Paz, 28029 Madrid, Spain; 5 CIBER de Enfermedades Respiratorias (CIBERES), Instituto de Salud Carlos III, 28029 Madrid, Spain; 6 Unidad Asociada de Biomedicina CSIC-UCLM, 02006 Albacete, Spain

**Keywords:** Transcriptome classification, Hypoxia, Gene expression, RNA-seq, Spatial transcriptomics

## Abstract

Molecular gene signatures are useful tools to characterize the physiological state of cell populations, but most have developed under a narrow range of conditions and cell types and are often restricted to a set of gene identities. Focusing on the transcriptional response to hypoxia, we aimed to generate widely applicable classifiers sourced from the results of a meta-analysis of 69 differential expression datasets which included 425 individual RNA-seq experiments from 33 different human cell types exposed to different degrees of hypoxia (0.1–5%$\hbox {O}_{2}$) for 2–48 h. The resulting decision trees include both gene identities and quantitative boundaries, allowing for easy classification of individual samples without control or normoxic reference. Each tree is composed of 3–5 genes mostly drawn from a small set of just 8 genes (EGLN1, MIR210HG, NDRG1, ANKRD37, TCAF2, PFKFB3, BHLHE40, and MAFF). In spite of their simplicity, these classifiers achieve over 95% accuracy in cross validation and over 80% accuracy when applied to additional challenging datasets. Our results indicate that the classifiers are able to identify hypoxic tumor samples from bulk RNAseq and hypoxic regions within tumor from spatially resolved transcriptomics datasets. Moreover, application of the classifiers to histological sections from normal tissues suggest the presence of a hypoxic gene expression pattern in the kidney cortex not observed in other normoxic organs. Finally, tree classifiers described herein outperform traditional hypoxic gene signatures when compared against a wide range of datasets. This work describes a set of hypoxic gene signatures, structured as simple decision tress, that identify hypoxic samples and regions with high accuracy and can be applied to a broad variety of gene expression datasets and formats.

**Supplementary Information:**

The online version contains supplementary material available at 10.1186/s12859-022-04741-8.

## Introduction

A gene expression signature is a single or combined group of genes whose expression is altered in predictable way in response to a specific signal or cellular status. Gene signatures are often derived from the set of differentially expressed genes (DEGs) identified when comparing two groups of transcriptomes, such as disease versus healthy controls or treated versus untreated samples. In turn, a gene signature can be of aid in trying to determine whether a given biological sample was exposed to that particular stimulus or belongs to the status defined by the gene set. Thus, reliable gene signatures can be used as surrogate markers for the activation of pathways or cellular status.

Hypoxia can be defined as the situation were oxygen supply does not meet cellular demand [1]. In response to hypoxia cells activate a gene expression program, under the control of the Hypoxia Inducible Factors (HIFs) [2], that aims to increase oxygen supply while reducing its consumption. Thus, this transcriptional response restores oxygen balance and, as such, it is central in maintaining tissue homeostasis. Importantly, oxygen homeostasis is disrupted in a number of prevalent pathologies including neoplasms [3] and cardio-respiratory diseases [4]. For all this reasons, the development of a hypoxic gene signature could be of practical interest to identify cells or samples that had been exposed to hypoxia, and accordingly, a number of studies have published hypoxic gene signatures [2, 5–11]. However, in spite of their merit, in all these cases the gene signature was derived from a limited set of related tumoral samples, raising the question of their applicability in other contexts. On another note, in almost all the cases, the gene signature is just a set of genes without any additional information reflecting their relative importance or their expected expression levels under normoxic/hypoxic conditions, meaning that it is nearly impossible to classify an individual isolated sample as normoxic or hypoxic based solely in the identities of the genes in the signature.

Herein we describe tree-based classifiers that accurately identify hypoxic cells or samples based on their gene expression profile. The identification is absolute, meaning that it does not require a set of normoxic reference samples to sort out the hypoxic ones. Thus, it can be applied to interrogate a single isolated sample. Finally, although the classifier implicitly contains information about the relative importance of the genes in the signature and their expression levels in hypoxia, it is simple enough to be interpreted and applied without the need for sophisticated computational tools.

## Materials and methods

### RNA-seq data download and processing

Raw reads of 121 RNA-seq experiments used as validation sets were downloaded from Sequence Read Archive [12]. Pseudocounts for each gene were obtained with salmon [13] using RefSeq [14] mRNA sequences for human genome assembly GRCh38/hg38 and mouse genome assembly mm10 as references.

Read counts of 70 tumoral and healthy samples were downloaded from the TCGA data portal and transformed to counts per million.

Spatial gene expression datasets for 7 experiments were downloaded from 10X Genomics website [15–21]. Raw read counts were normalized with sctransform [22] following Seurat v4.0.4 [23] standard pipeline for analysis, visualization, and integration of spatial datasets. No variable regression was performed during sample preprocessing, clustering nor PCA/UMAP reductions.

### Generation of a classifier

To generate the classifier we made use of 425 transcriptomic profiles of hypoxia-exposed cells and their normoxic counterparts described in a recent study [24]. From the gene pseudocounts in each sample, we calculated each gene’s ranking percentile and used this information in downstream analyses. We used the R package randomForest [25] to perform feature selection and the R package rpart [26] to generate decision trees. Random Forest hyperparameters were selected by cross-validation accuracy across 100 iterations for each of the possible values, ending up with 10 as the number of features and 200 as the number of trees generated. After hyperparameter tunning, the top genes were then selected by their “importance”, measured as mean decrease in accuracy (MDA) across 1000 iterations, resulting in a list of 20 genes used to generate individual decision trees. Each decision tree was evaluated by cross-validation using 70% of the available RNA-seq experiments as a training set and the remaining 30% as a validation set.

By default, a sample is classified as hypoxic when the tree assigns it a probability over 50% of being hypoxic, even though this threshold can be made stricter or laxer. The full collection of decision trees, validation data, and tutorial are available at www.github.com/LauraPS1/Hypoxia_Classifier.

## Results

### Generation of a hypoxic classifier

Results from our previous work [24] on differential expression triggered by hypoxia indicate that, even for those genes showing a significant regulation in the ensemble of datasets, the response to hypoxia could be in large part cell-specific. Thus, we sought to identify a minimal set of genes that could be used as a reliable readout of exposure to hypoxia and to develop a simple, easy to use, classifier that could identify whether an individual sample is hypoxic based on its gene expression.

**Fig. 1:**
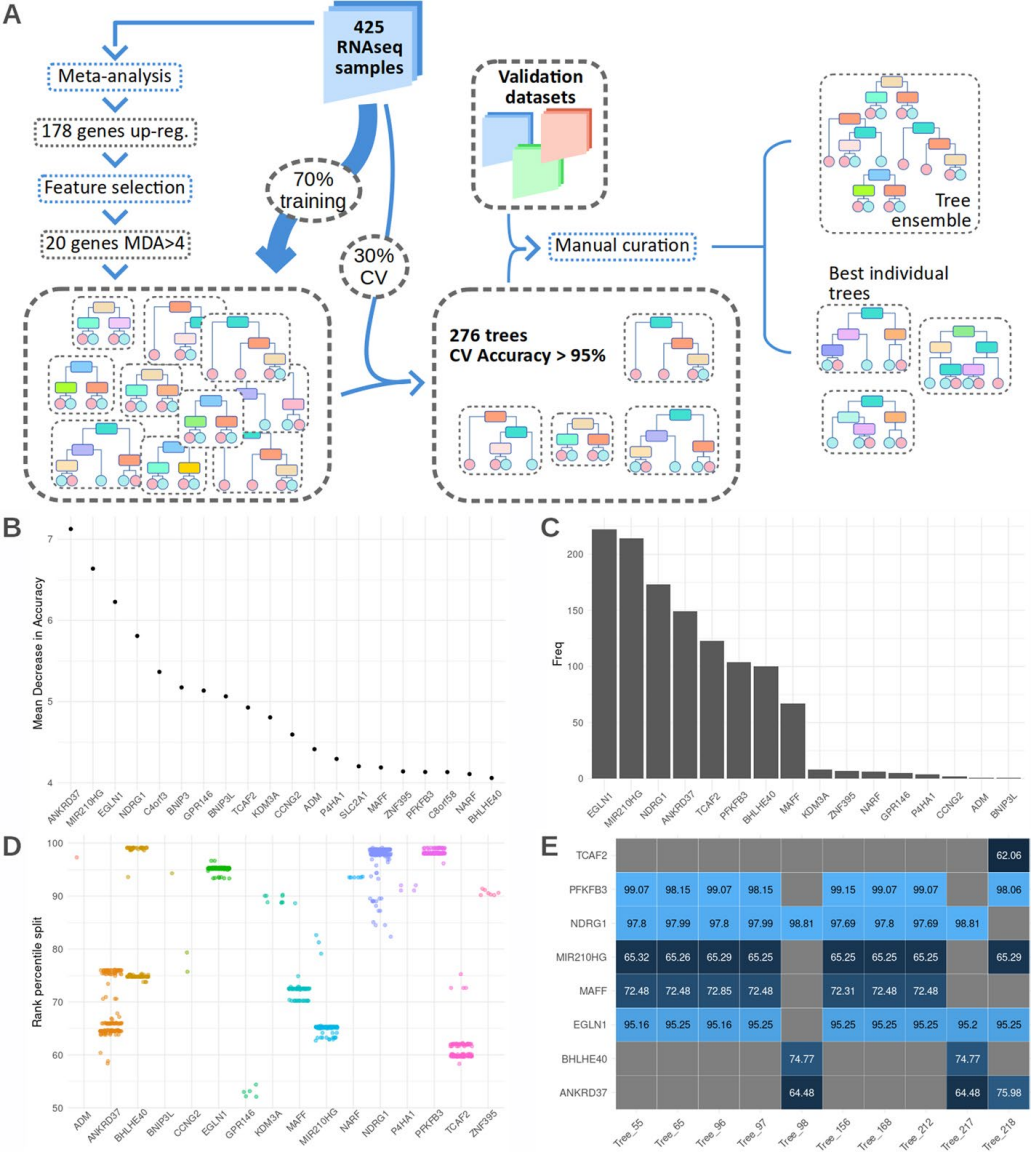
Generating expression based tree classifiers to identify hypoxic samples. **A** Decision trees generation overview. 425 RNA-seq samples exposed to normoxia or hypoxia were processed to produce a ranking set of genes from each of one them. A subset of the resulting data matrix, consisting in the 178 genes significantly up-regulated by hypoxia according to ref [24], was used as input to a feature selection algorithm. The 20 genes showing an MDA>4 were then selected to generate 10000 random trees and the 276 trees showing an accuracy over 95% in cross validation were selected as classifiers. Finally, a set of challenging datasets not used in the generation nor training steps, were used to test the performance of the 276 trees and select the best overall tree and two additional substitutes. **B** mean decrease in accuracy index of the 20 most important genes according to 1000 random forest iterations. **C** Frequency of each gene being used as a predictor variable in the classification trees. **D** Split points for the rank percentile (100 being the most expressed gene, 0, the least) of the genes used in all the models with accuracy > 0.95. **E** Split points for the rank percentile of the genes used in the 10 best performing models according to cross-validation accuracy

With the goal of making the model as widely applicable as possible we chose to use as input data the percentile of each gene on a gene expression ranking, thus minimizing the effects of read depth, different normalization methods, possible rRNA contamination, and other factors that influence RNA quantification. Thus, we first constructed a gene ranking matrix for a set of 425 individual RNA-seq samples derived from published transcriptomic analysis of hypoxic cells and controls (Fig. 1A). For subsequent analyses we kept the subset of 178 genes both significantly up-regulated by hypoxia ($LFC>0.7$, $FDR<0.01$) and widely expressed (detectable in $\ge 90\%$ of the analyzed subsets) according to a meta-analysis performed on this data set [24].

In order to select the most informative genes in this subset, we used 1000 iterations of a random forest classifier sampling 70% of the RNA-seqs at each iteration (Fig. 1A). As a measure of each gene’s importance we use the mean decrease in accuracy (MDA), representing how much accuracy the model losses by excluding each gene, across all iterations. The 20 genes with average MDA over 4 (Fig. 1B) were selected to train 10000 decision trees randomly sampling 70% of the individual RNA-seq experiments and using the remaining 30% as a validation set. The 276 trees with an accuracy over 0.95 on the validation set were selected to further test their performance (Additional file 1: Table S1 sheet 4, “Cross validation” and Fig. 1A).

Only 16 genes are used in all of the 276 decision trees, with half of them (EGLN1, MIR210HG, NDRG1, ANKRD37, TCAF2, PFKFB3, BHLHE40, and MAFF) being included overwhelmingly more frequently (Fig. 1C). Most of these genes have already been linked to the transcriptional response to hypoxia [27–34], even though in some cases their particular role in it has not yet been defined.

In these classification trees, at each node, the algorithm evaluates the rank percentile of the pertinent gene to determine which branch will be followed for the classification of the sample, hence the final sample label is assigned based on the relative (percentile rank) expression values of the genes in the tree. As seen in Fig. 1D, the split point for most genes is limited to a narrow range of rank percentiles, with ANKRD37 and BHLHE40 being the exception. These two genes show two differentiated split points that depend on the identity of remaining genes in the tree: for ANKRD37, it depends on whether its combined together with NDRG1 or TCAF2, while BHLHE40’s depends on whether the tree includes MIR210HG. Fig. 1E represents gene identity and split points for the 10 best trees according to cross-validation accuracy. Both in this top 10, as well as in the whole set of 276 trees we find a limited number of topologies present, with MIR210HG, EGLN1, MAFF, NDRG1, and PFKFB3 forming the most common combination, closely followed by ANKRD37, NDRG1, and BHLHE40.

### Evaluation and validation of the resulting decision trees

In order to evaluate the performance of each one of the 276 decision trees with an accuracy over 95% and test particular strengths and weaknesses of each model, we tested them on a series of datasets that were not part of the training nor cross-validation sets and had some differential feature that posed a challenge to the classification (Additional file 1: Table S1 sheet 2 “RNA-seq metadata”). In addition to evaluate their performance, the result of these analyses guided us in the selection of the trees best suited to be used as general and robust hypoxia classifiers.

**Fig. 2:**
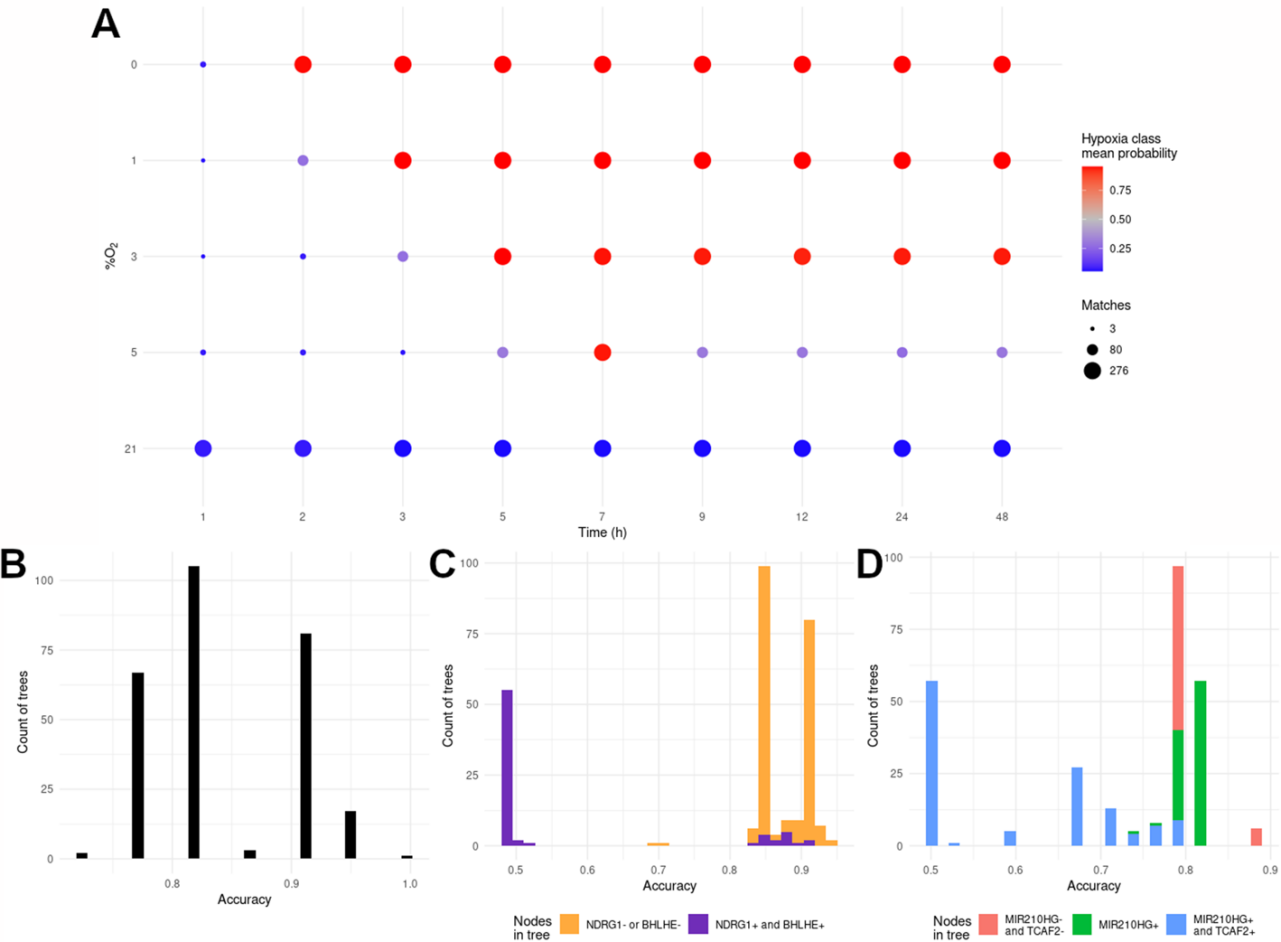
Validation of tree classifiers on novel datasets. **A** Time and oxygen series dataset. Each of the dots represents one of the samples in the five time series in PRJNA561635, ordered by oxygen tension and time. The color of each dot represents the mean probability of each sample to be classified as hypoxic, while the size of the dot is proportional to the number of trees correctly classifying each sample. **B**–**D** Distribution of the accuracy of the 276 classification trees on validation datasets. Different colors are used to identify trees including specific genes and/or topology as indicated in the legend. See text for details. **B** 22 datasets from RNA fractions other than total mRNA. **C** 2 datasets of clear cell renal carcinoma with VHL mutations and paired adjacent healthy tissues. **D** 34 mouse RNA-seq datasets

First we chose a time series experiments available on PRJNA561635 [35], consisting of a set of transcriptional profiles of Human Umbilical Vein Endothelial Cells (HUVEC) exposed to different oxygen concentrations at nine time points. The main challenge with this validation set is detecting early stages of hypoxia (1–3 hours), where most hypoxia-target genes have just barely began to accumulate, and differentiate mild hypoxic stress (3% oxygen) from physoxia (5% oxygen), which is within the range of physiological oxygen concentration found in many tissues [36] and hence *in vitro* trigger a weaker transcriptional response for many genes [37]. As shown in Fig. 2A and Additional file 1: Table S1 sheet 5 “PRJNA561635”, all decision trees correctly classified normoxic samples and samples exposed to oxygen levels at or below 3%O_2_ (i.e. physiological hypoxia, [36]) for at least 5h. It is worth highlighting that around a third of the trees were also able to detect earlier stages of hypoxia (2h 1%O_2_, 3h 3%O_2_). In addition, these results clearly show that the lower the oxygen tension, the strongest the signal detected at early times of exposure with 5% oxygen being at the boundary between normoxia and physiological hypoxia.

The next validation set consisted of four studies on specific fractions of RNA: newly transcribed RNA(4sU labeling RNA-seq and GRO-seq [38, 39]) and actively translated RNA (polysomal RNA-seq [40]) (Additional file 1: Table S1 sheet 6, “RNA fractions”) [41–43]. In this case the challenge stems from the different RNA fractions used in the derivation of the trees (total mRNA) and test datasets. In spite of the different source of RNA, all decision trees had an accuracy over 0.75, with 36% showing an accuracy over 0.9 in the classification of 4sU labeling, GRO-seq, and polysomal samples (Fig. 2B).

The transcriptomic response to low oxygen tension can be induced by specific genetic lesions even under normoxia, so next we tested whether the classification trees could identify such samples, in spite of being derived from cells not exposed to hypoxia. Specifically we tested whether they could differentiate between clear cell renal carcinoma samples (ccRCC) and paired healthy adjacent tissues (Additional file 1: Table S1 sheet 7, “ccRCC”) from both the TCGA-KIRC collection and another publicly available study [44]. Over 80% of ccRCC show mutations in the von Hippel-Lindau (VHL) gene that encodes for a key molecule controlling HIF stability. Thus VHL mutation leads to chronic HIF activation, even in the presence of oxygen, leading to a hypoxia-like transcriptional pattern [45, 46]. As shown in Fig. 2C, the vast majority of the trees, 216 out of 272, were able to identify VHL-mutant cells with an accuracy over 83%, even though no ccRCC samples were included in the training nor cross-validation datasets.

Finally, since the tree-classifiers were derived from human samples, we decided to test its performance on transcriptomes from other organisms. To that end, we gathered five studies in murine cells, totalling 34 individual RNA-seq experiments performed in different cell types and experimental conditions (Additional file 1: Table S1 sheet 2 “RNA-seq Metadata”) [47–51]. As in the previous cases, the majority of trees (160 out 276) were able to classify samples with an accuracy of 79% or higher (Fig. 2D).

Altogether these results indicate that the tree-classifiers show a remarkable performance on novel datasets not used during the generation nor training steps, and correctly identify hypoxic samples derived from a wide range of conditions outside those represented in the training set. In spite of this, the bimodal distribution observed in Fig. 2C and 2D, suggest that a subset of the trees did not behave well on specific datasets. Closer analysis of these cases revealed that of the 58 trees showing poor performance against the ccRCC datasets, 42 share a common structure that includes only three genes organized in two levels, with ANKRD37 being the root nodes and two branches, one evaluating NDRG1 and the other BHLHE40 (Additional file 4: Fig. S1A). Although the expression of ANKRD37 differs in both groups (Additional file 4: Fig. S1B), both NDRG1 and BHLHE40 are already highly expressed in normal kidney samples (Additional file 4: Fig. S1C and D), explaining why trees with this topology are unable to differentiate between conditions. In the case of mouse datasets, we found that most of the best classifiers did not included MIR210HG in their structure (Fig. 2D), which stands to reason as there are no MIR210H orthologs annotated in mouse, therefore this feature does not convey relevant information for the classification in this case.

**Fig. 3:**
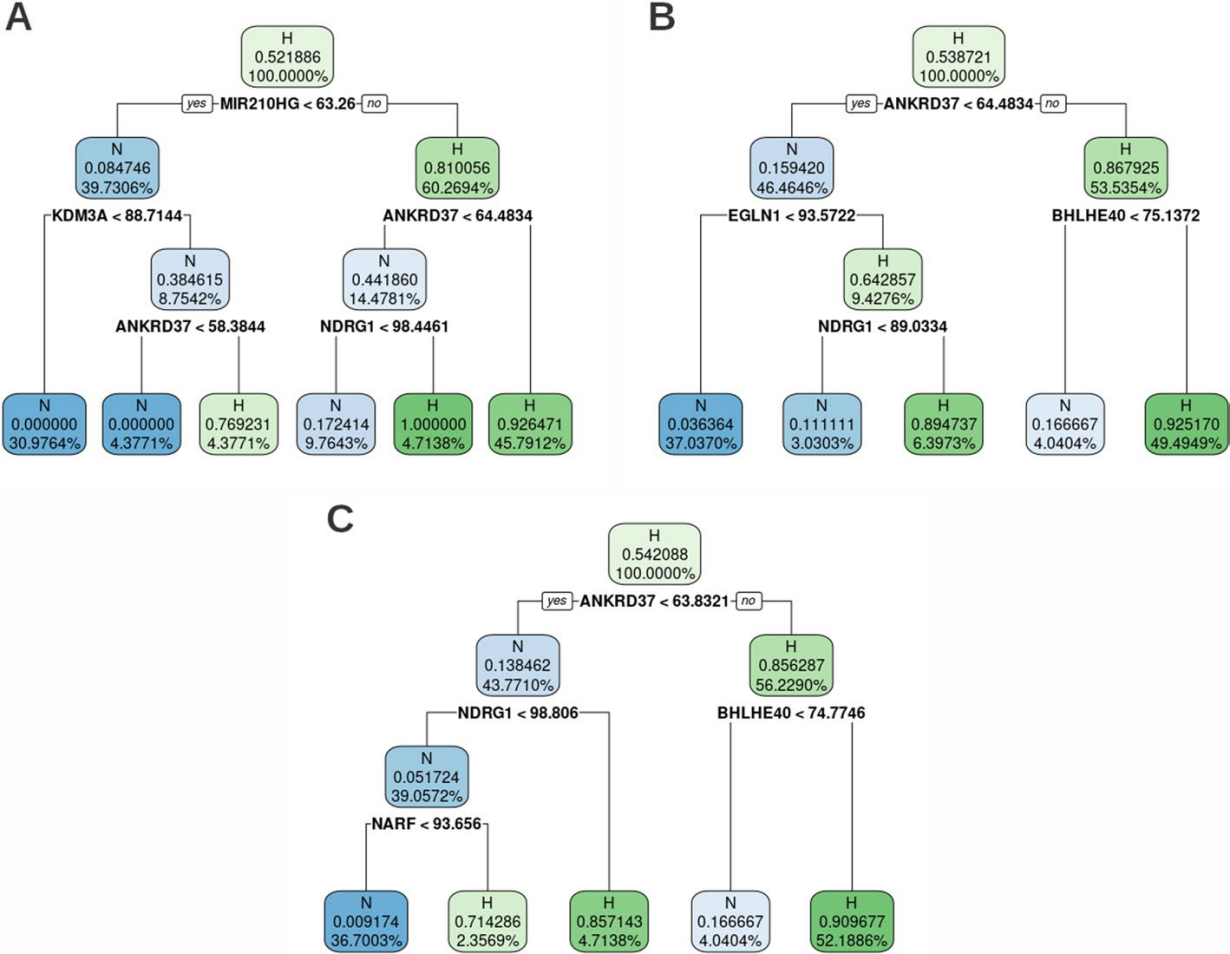
Selected decision trees. The labels in each node indicate the node’s class (N, normoxic, and H, hypoxic), the probability of samples in the node to be classified as hypoxic, and the percentage of samples of the training set in each node. Each node is colored according to the probability of samples in said node to be classified as hypoxic. **A** Overall best performing tree (tree #125). **B** Best performing tree among those not using MIR210HG (tree #241) **C** Best performing tree on mouse data (tree #42)

Altogether the analyses presented above allowed us to identify a subset of trees that accurately classify samples even from challenging datasets. Fig. 3A shows the best performing tree overall, especially apt in detecting short exposure to hypoxia and mildly low oxygen levels without overestimating the number of hypoxic samples in other validation sets. Since specific RNA types such as lncRNAs and microRNAs might not be represented in all sequencing libraries, we also selected the tree in Fig. 3B, being the best performing among those that don’t include MIR210HG lncRNA gene. Even though both trees perform reasonably well on mouse data, we have selected the additional tree in Fig. 3C for being the best performing in classifying murine samples specifically.

**Fig. 4:**
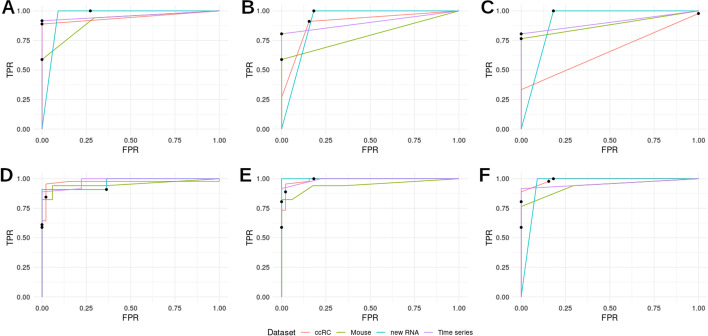
Tree ensembles performance. ROC curves for individual trees and three ensembles using four validation datasets. A sample was classified as hypoxic when the mean probability given by the trees surpassed a given threshold between 0 and 1. Black dots represent TPR/FPR values for a probability threshold of 0.5 to classify a sample as hypoxic. **A**–**C** ROC curves for the individual trees selected for their performance. **A** Tree #125 (Fig. 3A). **B** Tree #241 (Fig. 3B). **C** Tree #42 (Fig. 3C). **D**–**E** ROC curves for three tree ensembles. **D** all 276 trees. **E** top 20 trees by medium F_1_-score. **F** three selected trees (#125, #241, #42)

Since the selected trees derive from manual curation against a limited set of conditions outside those in the original datasets and it is unlikely that a single tree could accurately classify samples from all potential datasets, we tested whether classification would improve using several trees and generating a consensus. To this end, we compared the performance of the individual three selected trees (Fig. 4A–C) against three ensembles: the whole 276 collection (Fig. 4D), the 20 trees with higher mean F_1_-score (Fig. 4E) and the aforementioned three selected trees (Fig. 4F). AUC values for each curve are displayed in Table 1. The result of this analysis shows that, as expected, the ensembles tend to outperform individual trees. However, the difference is small and in some datasets individual datasets performed as well as the ensembles.

**Table 1:** Area under the curve corresponding to ROC curves for individual trees and three ensembles using four validation datasets. A sample was classified as hypoxic when the mean probability given by the ensemble or individual tree surpassed a given threshold between 0 and 1

Model	Time series	RNA fractions	ccRC	Mouse
Ensemble 276 trees	0.978	0.967	0.969	0.953
Ensemble top 20	0.991	1.000	0.990	0.946
Ensemble selected 3	0.958	0.955	0.990	0.936
Tree 125	0.958	0.955	0.944	0.910
Tree 241	0.903	0.909	0.899	0.794
Tree 42	0.903	0.909	0.656	0.882

Thus, we have constructed a classification tree (tree #125, Fig. 3A,) that based on the ranked expression of just four genes (MIR210HG, KDM3A, ANKRD37 and NDRG1) is able to correctly identify normoxic/hypoxic samples with an accuracy of over 95% and 0.96 F_1_- score. The robustness of the predictions can be further improved by using the consensus decision from more than one tree.

### Application of the classifier to identify hypoxic tumors and hypoxic regions within tissues

**Fig. 5:**
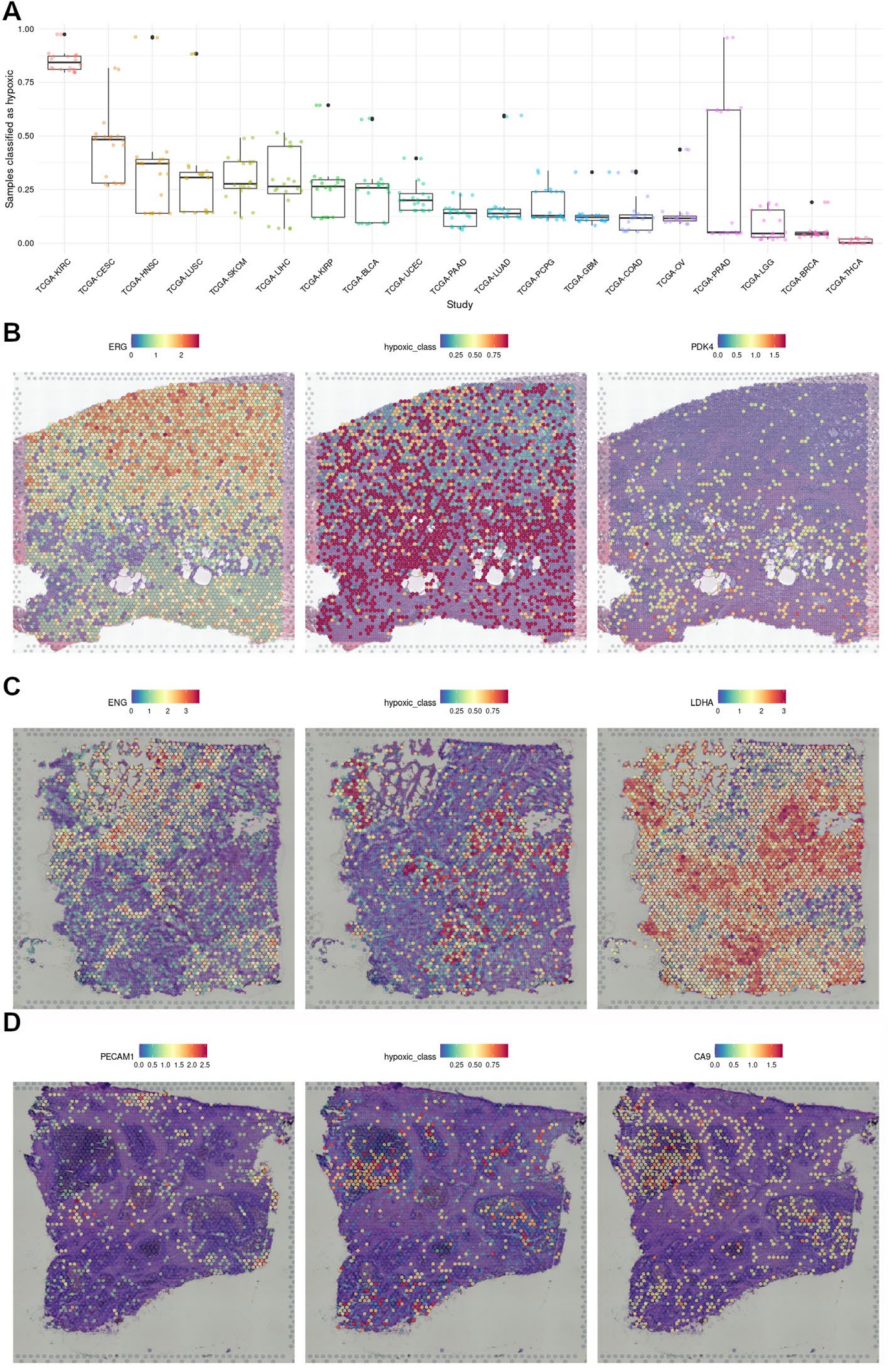
Detection of hypoxia in TCGA transcriptomes and tumor sections. **A** Proportion of TCGA tumor samples classified as hypoxic by an ensemble of the 20 decision trees with higher F_1_-score, by primary site. **B**–**D** Spatial Gene Expression datasets were downloaded from from 10X Genomics and used as input for the tree classifiers to detect hypoxic regions. On the left column, the expression of ENG, and PECAM1 are highlighted as endothelial markers, while on the right the expression of PDK4, LDHA, and CA9 mark regions of active anaerobic glycolysis. The central column represents the probability of each spot to be classified as hypoxic. **B** Human Prostate Cancer, Adenocarcinoma with Invasive Carcinoma (FFPE). **C** Human Glioblastoma. **D** Human Colorectal Cancer

Most solid tumors are hypoxic due to their aberrant growth and vascularization. Given that the presence of hypoxia compromises cancer therapy and is a poor prognosis factor, the identification of hypoxic tumors is relevant to predict tumor progression and select appropriate treatment strategies [52]. Thus, we next studied the ability of the tree classifier to identify hypoxic tumors. To that end we applied the classifier to transcriptomic profiles from The Cancer Genome Atlas (TCGA) and determined, for each type of tumor, the proportion of samples classified as hypoxic. As shown in Fig. 5A, the tumor type with the highest proportion of cases classified as hypoxic is the Kidney Renal Clear Cell Carcinoma (KIRC), in agreement with the molecular alterations characteristic of this cancer. Moreover, although the ranking of tumors varies across studies [52, 53], head and neck and cervix carcinomas tend to be very hypoxic tumors as determined by direct measure of pO_2_ using oxygen electrodes, also in agreement with the classifier prediction shown in Fig. 5A.

One of the challenges in the study of tumor hypoxia is the heterogeneity of oxygenation within the tumoral mass [36]. The identification of hypoxic areas within a tumor typically relies on the detection of a single or a few markers of hypoxia such as the presence of HIFs or HIF targets [52]. The availability of spatial transcriptomic datasets allows for the identification of tissue hypoxia based on a gene signature rather than a single marker, so we decided to take advantage of the availability of several spatially resolved tumor sample transcriptomes [15, 17, 18] to test the ability of the tree classifiers to identify hypoxic regions in glioblastoma, prostate, and colorectal cancer. Each spot in the samples was classified as normoxic/hypoxic applying an ensemble of the 20 trees with higher mean F_1_-scores across validation datasets. For datasets that did not include MIR210HG expression, we generated an ensemble with trees that do not require this gene’s expression value.

This analysis revealed that regions identified as hypoxic by the tree classifiers, correspond to those poorly vascularized, according to vascular markers, and expressing high levels of glycolytic enzymes (Fig. 5B–D). It is worth mentioning that none of these reference markers were previously used to evaluate the performance of the classifiers.

**Fig. 6:**
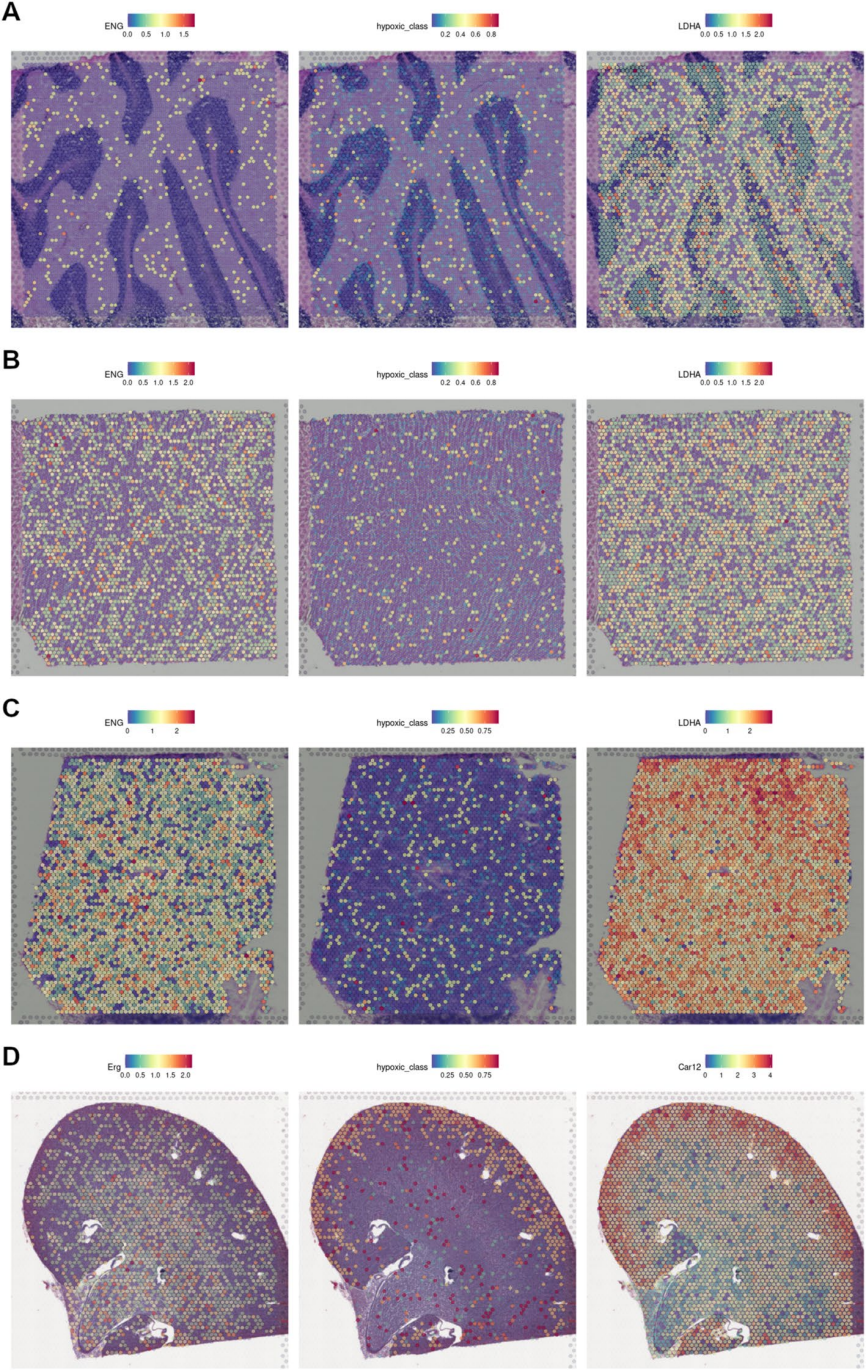
Detection of hypoxic regions within normal tissue sections. Spatial Gene Expression datasets were downloaded from from 10X Genomics and used as input for the tree classifiers to detect hypoxic regions. On the left column, the expression of ERG, ENG are highlighted as endothelial markers, while on the right the expression of Car12, LDHA mark regions of active anaerobic glycolysis. The central column represents the probability of each spot to be classified as hypoxic. **A** Cerebellum. **B** Heart. **C** Lymph node. **D** Kidney

In sharp contrast to the pervasive presence of hypoxic areas in most tumors, normal tissues usually do not show detectable HIF activity [54]. In order to test the specificity of the hypoxic signal detected by our classifiers, we next analyzed the spatial transcriptomes of normal tissues [19–21]. As shown in Fig. 6, with the exception of the kidney cortex, none of the normoxic tissues presented defined normoxic areas. These results are in agreement with a report showing that the kidneys are the only organ in showing HIF activity in normal mice breathing room air [55]. As further confirmation we proceeded to cluster the spots in each dataset (Additional file 5: Fig. S2) and examined differential expression between clusters containing high or low proportion of spots classified as hypoxic (Additional file 2: Table S2–9 and Additional file 6: Fig. S3).

Altogether, these results support the utility of our classifiers beyond bulk RNA-seq datasets, considering they accurately identify hypoxic tumoral samples and hypoxic regions in spatial gene expression datasets.

### Comparison with previously published hypoxia gene signatures

**Fig. 7:**
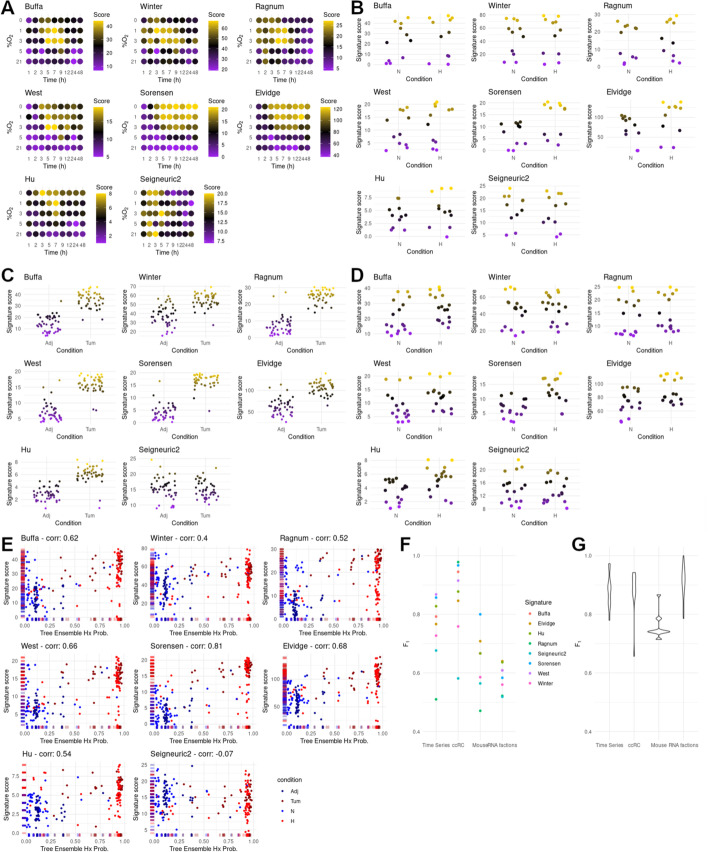
Performance of published hypoxic gene signatures. **A**–**D** Application of 8 hypoxia gene signatures as described in [56] to training and validation datasets. **A** PRJNA561635 time series. **B** Specific RNA fractions other than total mRNA. **C** ccRCC tumor and healthy adjacent tissue samples. **D** mouse datasets. **E** Correlation between the hypoxia scores derived from the 8 molecular signatures tested and the hypoxia probability calculated using an ensemble of our 20 best classifiers calculated for all samples from the validation experiments. Color code goes as follows; blue: samples grown in normoxia, red: samples grown in hypoxia, dark blue: healthy kidney samples, dark red: ccRCC tumoral samples. **F**–**G** distribution of F_1_-scores for normal and tumoral samples from the validation datasets using the published gene signatures and tree classifiers. **F** Molecular signatures used in [56]. Samples were classified as hypoxic when the score calculated exceeded 50% of the maximum for that dataset and signature. **G** 20 individual trees that composed the ensemble. Samples were classified as hypoxic when the probability given by a tree exceeded 0.5

As indicated before, a number of hypoxic gene signatures have been previously described [2, 5–11], most of them derived from the lists of DEGs in response to hypoxia in specific tumors. Although these signatures are mostly defined as mere lists of genes and, as such, cannot be used to classify samples, Bhandari and co-workers [56] described a method to derive an hypoxic score value based on these lists of genes. Unlike the tree classifiers described herein, this score can not identify a sample as being hypoxic or normoxic, however, it allows the relative comparison among samples. We made use of this hypoxic scoring method to assess the relative ability of the individual gene signatures to discriminate between normoxic and hypoxic samples in the validation datasets described above (time series, RNA fractions, ccRCC and mouse RNA-seq datasets). Fig. 7A shows that the performance of the different gene signatures on the time series dataset varies widely and that only the scoring based on the Sorensen signature [9] results in a relative separation of samples that resembles their true labels. In the case of the different RNA fractions datasets, all gene signatures perform poorly as demonstrated by the very similar distribution of hypoxic scores assigned to normoxic and hypoxic samples (Fig. 7B), with only around 60% of the hypoxic samples having a score above those assigned to normoxic samples in the best cases. In contrast to these results, most signatures resulted in a good relative classification of normal and tumoral samples from the ccRCC datasets, as indicated by the score of the tumoral samples being higher than that of normal kidney ones (Fig. 7C). In spite of this, there was a substantial overlap between the two groups of samples for some signatures (Winter, Elvidge and Seigneuric2). Finally, we tested the gene signatures against samples from mouse cell lines, and as shown in Fig. 7D, even the best performing signatures (Sorensen and Elvidge), were unable to assign a score above controls to the majority of the hypoxic samples. Next, to directly compare the performance of the tree classifiers with the aforementioned gene signatures, we represented the hypoxic score assigned by each gene signature against the probability assigned by the ensemble of the 20 best trees for all the samples included in the validation datasets (time-course, RNA fractions, ccRCC and mouse RNA-seq samples). Fig. 7E shows that, although the two measures correlate for most gene signatures, the tree-based classifier described herein outperforms all gene signatures as evidenced by the better separation of samples according to the X-axis than the Y-axis. Finally, Figs. 7F–G compare the performance of individual trees and gene signatures against each validation dataset. Remarkably, in the case of the most favorable dataset (clear cell renal carcinoma), individual trees perform similarly to the best gene signatures while thoroughly outperforming them in the rest of validation datasets.

As a whole these results indicate that, in contrast to our classifiers, most of the published hypoxic gene signatures are less reliable when identifying cells exposed to hypoxia outside of the biological context each signatures was developed in. Basing our classifiers on the results of an extensive meta-analysis grants them the degree of flexibility needed to maintain accuracy against new data and different biological contexts.

## Discussion

In this work we aim to derive a gene signature that, besides defining the minimum core of genes that characterize the response to hypoxia, could be used to assess if an individual gene expression dataset corresponds to sample that has been exposed to low oxygen tension. Additionally, one of the main priorities in the design of this classifier was to keep maximum transparency and interpretability in the process, so that, with a minimal or no background in machine learning, any user can not only determine if their sample is hypoxic, but also trace why it was marked as hypoxic.

This work is based on a meta-analysis of the transcriptomic response to hypoxia, generated through the integration of a corpus of 69 differential expression datasets which included 425 individual RNA-seq experiments from 33 different cell types exposed to different degrees of hypoxia (0.1–5%$\hbox {O}_{2}$) for a period of time spanning between 2 and 48 h [24]. As a first filter of the variables (genes) to be included in the signature, we selected those widely expressed and significantly up-regulated by hypoxia according to the meta-analysis. This step ensured that the resulting models can be applied to a large variety of tissues as well as minimizing the risks of a biased corpus of publicly available experiments. Then we applied data mining methods to identify sets of genes that best separated normoxic and hypoxic samples using a tree-like decision structure. Although the total number of trees that achieved high accuracy was relatively large, only 16 out of the 20 pre-selected genes were required among all the trees, with many having the same structure and differing only slightly in the gene expression threshold. Moreover, the vast majority of trees included different combination of 3–5 genes from the set EGLN1, MIR210HG, NDRG1, ANKRD37, TCAF2, PFKFB3, BHLHE40, and MAFF (Additional file 1: Table S1).

In contrast with classical molecular signatures, the trees described herein provide not just a list of genes relevant to the process, but also a set of matching quantitative expression boundaries, which allows it to classify individual samples both from a binary perspective (hypoxic or normoxic sample) as well as a continuous one (probability of a sample to be classified as hypoxic, shown in Figs. 5B–D and 6). The features of the classifiers permit their application of the classification trees to a wide range of gene expression datasets, from the conventional bulk RNA-seq by polyA capture and techniques to characterize newly transcribed RNA [38, 39] to spatially resolved transcriptomics and single cell RNA-seq. Importantly, gene’s expression boundaries are represented as the percentile occupied by the gene in a ranked list of expression values from a given sample, which means that this method can be applied to a diverse set of input formats: raw reads, counts per million, FPKM, variance stabilizing transformations, etc.

It should be noted that these classifiers are robust enough to predict the condition of samples from murine cells despite being trained only on human datasets, as well as identify samples in which response to hypoxia is activated by mutations in specific signalling pathways (ccRCC dataset), due to the pattern of vascularization and/or oxygen consumption (TCGA datasets, tumor specimens) and even hypoxic regions present in normal tissues (kidney dataset).

In regard to the classification of tumor samples according to their degree of hypoxia, our results are in good agreement to those reported in [56] using different hypoxic signatures (Additional file 1: Table S1 sheet 8). However, unlike the tree classifiers described herein, other signatures failed to identify clear cell renal carcinomas as the type of tumor showing the highest up-regulation of the hypoxic transcriptome [56]. Nevertheless, as shown in Fig. 5A, with the exception of renal carcinomas, the proportion of tumor samples classified as hypoxic in each group resembles more closely the results obtained by Bhandari et al. [56]. On the other hand, previously reported hypoxic signatures performed poorly against non-tumoral validation datasets described in our work, as shown in Fig. 7 and Additional file 3: Table S3. Considering that all but one of the classic gene signatures were defined in the context of tumor hypoxia, a poor correlation could be expected when compared to the performance of a classifier trained with a more diverse corpus of experiments (Fig. 7E).

As further confirmation of the effectiveness of the trees in comparison to classic gene signatures, we decided to test them in spatial gene expression datasets, using the expression levels of endothelial markers ERG, ENG and PECAM1/CD31 to localize well oxygenated regions and the expression levels of genes related to anaerobic glycolysis, to define regions of restricted oxygen availability. As shown in Fig. 5B–D, regions classified as hypoxic overlap those of active anaerobic glycolysis, meanwhile regions rich in endothelial markers tend to be classified as normoxic. After unsupervised clustering of the same datasets (Fig. S2), differential expression between clusters overlapping normoxic and hypoxic areas highlighted genes linked to hypoxia and not included in our models, such as VEGFA or ENO1 (Additional file 2: Table S2). Furthermore, when comparing the adjusted *p*-values of genes up-regulated between clusters that are also significantly up-regulated in the cited hypoxia meta-analysis [24] (random effect>0.7 and FDR<0.01) this group has significantly lower p-values than genes not linked to hypoxia, confirming an enrichment on hypoxia-related genes among those differentially expressed between hypoxic and normoxic clusters. In contrast to the results obtained with tumor sections, we did not found significant hypoxic regions in normal tissues (Fig. 6). Which is consistent with the absence of HIF activation in tissues under physiological conditions [54], in spite the wide range of pO_2_ values found in normal tissues [36]. The only exception was the identification of the kidney cortex as an hypoxic region (Fig. 6), which, although unexpected at first glance, is in agreement with the results from a noninvasive imaging technique that identified the kidneys as the only organ in showing HIF activity in normal mice breathing room air [55]. Moreover, although pO_2_ in the medulla is lower than in the cortex, the renal medulla presents a comparatively higher expression of the HIF inhibitors EGLNs [57], which might explain why no constitutive HIF stabilization is found in the medulla under physiological conditions [58] and thus why this region is not labeled by the tree classifier.

In addition to their remarkable performance, the structure of the decision trees allows for biological interpretation of the prediction’s results. In this regard, the application of the decision trees to the challenging datasets provided relevant and novel insights into the underlying biological processes. For example, the analysis of the performance of different trees on the ccRCC dataset revealed that BHLHE40 and NDRG1 are expressed at high levels in renal tissue which can hint to specific functions of these genes in kidney physiology. On the other hand, as seen with the mouse dataset in Fig. 2D, missing data in one of the classifying variables (MIR210HG) could directly or indirectly hinder the performance of the trees. Thus, we tested if performance of the tree classifiers can be improved by generating a consensus. As we show in Fig. 4, an ensemble of the 20 trees with higher mean $\hbox {F}_{1}$-score (Fig. 4D) can outperform all individual trees and other ensembles in most cases (with the exception of tree #42 in the mouse dataset). A classification based just in the consensus of the three trees selected in this paper (Fig. 4F) can compensate for the shortcomings of each individual model while maintaining the ease of use intended for this classifier. Tree ensembles could be a better suited alternative for samples that are harder to classify or derived from a dataset distantly related to the ones used to derive our tree classifiers.

In summary, herein we describe a ensemble of tree gene signatures that can be easily implemented to identify hypoxic samples based on their transcriptomic profile without the need for a reference. Given the importance of oxygen homeostasis in physiology and disease, this tool could be useful in a wide variety of research and clinical settings. Finally, in the view of its merits, we proposed the extension of this method to define gene signatures that characterize other cellular processes.

## Supplementary Information

**Additional file 1: Table S1.** Classification tree validation. Sheet 1: metadata for the experiments used for model generation. Sheet 2: metadata for each of the experiments used in the validation process. Sheet 3: summary of tree variables and validation accuracy. Sheets 4-7: performance measurements for each human validation dataset. Sheet 8: Proportion of TCGA tumor samples classified as hypoxic by primary site. Sheet 9: performance measurements for the mouse validation dataset.

**Additional file 2: Table S2.** Differential expression in unsupervised clustering of spatial gene expression datasets: Differentially expressed genes between clusters containing a high proportion of spots classified as hypoxic and clusters with low or no hypoxic spots. Sheet 1: Prostate cancer, C7 vs C0. Sheet 2: Prostate cancer, C11 vs C2. Sheet 3: Mouse kidney, C3 vs C1. Sheet 4: Mouse kidney, C5 vs C6.Sheet 5: Glioblastoma, C12 vs C8.Sheet 6: Glioblastoma, C1 vs C5 . Sheet 7: Colorectal cancer, C14 vs C2. Sheet 8: Colorectal cancer, C7 vs C8. Sheet 9: Mann–Whitney test results for differences in p-values among genes up-regulated between clusters according to their up-regulation status in our hypoxia meta-analysis (random effect>0.7, FDR<0.01).

**Additional file 3: Table S3.** Comparison between tree classifiers and previously published hypoxia gene signatures. Application of the method described in [56] of 8 hypoxic gene signatures to the validation datasets. Each table includes, for every sample, the hypoxic score derived from each signature, as well as the probability of being hypoxic assigned by our ensemble of the 20 trees with higher F1-score. Sheet 1: PRJNA561635 time series. Sheet 2: specific RNA fractions other than total mRNA. Sheet 3: ccRCC tumor and healthy adjacent tissue samples. Sheet 4: murine datasets.

**Additional file 4: Fig. S1.** Identification and characterization of faulty classification trees in clear cell renal carcinoma samples. A: Representative example of a poorly performing tree. B-D: Ranking percentiles of genes common in poorly performing trees in the samples making up the clear cell renal carcinoma validation set. The red line represents the mean split point for each gene in the poorly performing trees. B: ANKDR37. C: NDRG1. D: BHLHE40.

**Additional file 5: Fig. S2.** Unsupervised clustering matches areas classified as hypoxic. UMAP representations (first column) and microscopy overlay (second column) of spot clustering using a shared nearest neighbor (SNN) based algorithm. Spots marked as hypoxic by the decision trees group together and ”hypoxic” clusters are positioned close by in the UMAP space. A: Human Prostate Cancer, Adenocarcinoma with Invasive Carcinoma (FFPE). B: Adult Mouse Kidney (FFPE). C: Human Glioblastoma. D: Human Colorectal Cancer.

**Additional file 6: Fig. S3.** Hypoxic genes DE between Visium dataset clusters. -Log10 (FDR adjusted p-values) of genes significantly up-regulated between clusters, according to their up-regulation status in the previously cited hypoxia meta-analysis. A: Prostate cancer, C7 vs C0. B: Prostate cancer, C11 vs C2. C: Mouse kidney, C3 vs C1. D: Mouse kidney, C5 vs C6.E: Glioblastoma, C12 vs C8.F: Glioblastoma, C1 vs C5 . G: Colorectal cancer, C14 vs C2. H: Colorectal cancer, C7 vs C8.

## Data Availability

References to the sources of experiments used as to generate the decision trees are stored in Additional file 1: Table S1, sheet 1. References for the validation datasets are included in Additional file 1: Table S1, sheet 2. The full collection of 276 decision trees, gene ranking percentiles for the validation datasets used, as well as a step-by-step guide to apply the classifiers to new data are available at the following GitHub repository: www.github.com/LauraPS1/Hypoxia_Classifier.
