# Supplementary figures and images for: Hypoxia classifier for transcriptome datasets
Source: BMC Bioinformatics. 2022 May 31;23:204. doi: 10.1186/s12859-022-04741-8 (PMC9153107; doi:10.1186/s12859-022-04741-8)

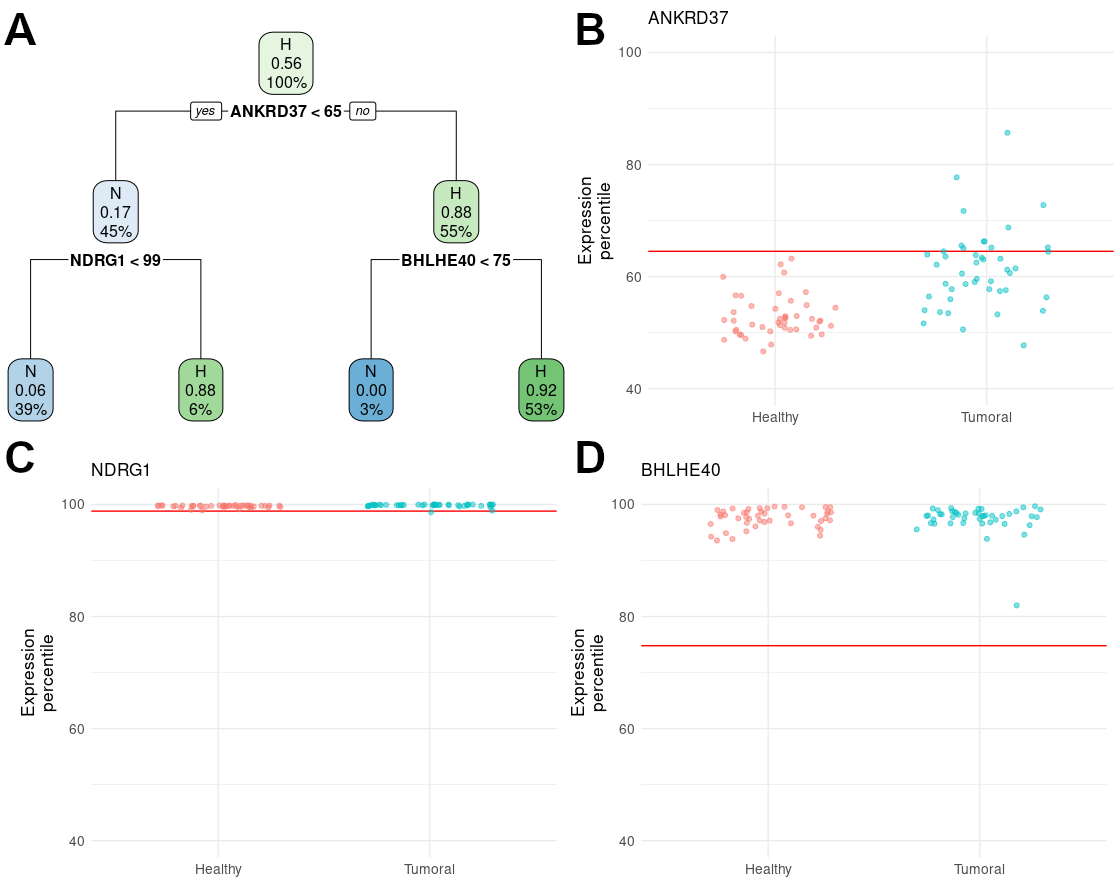

Supplement: Supplementary file 4 — Additional file 4: Fig. S1. Identification and characterization of faulty classification trees in clear cell renal carcinoma samples. A: Representative example of a poorly performing tree. B-D: Ranking percentiles of genes common in poorly performing trees in the samples making up the clear cell renal carcinoma validation set. The red line represents the mean split point for each gene in the poorly performing trees. B: ANKDR37. C: NDRG1. D: BHLHE40. [file 12859_2022_4741_MOESM4_ESM.png]

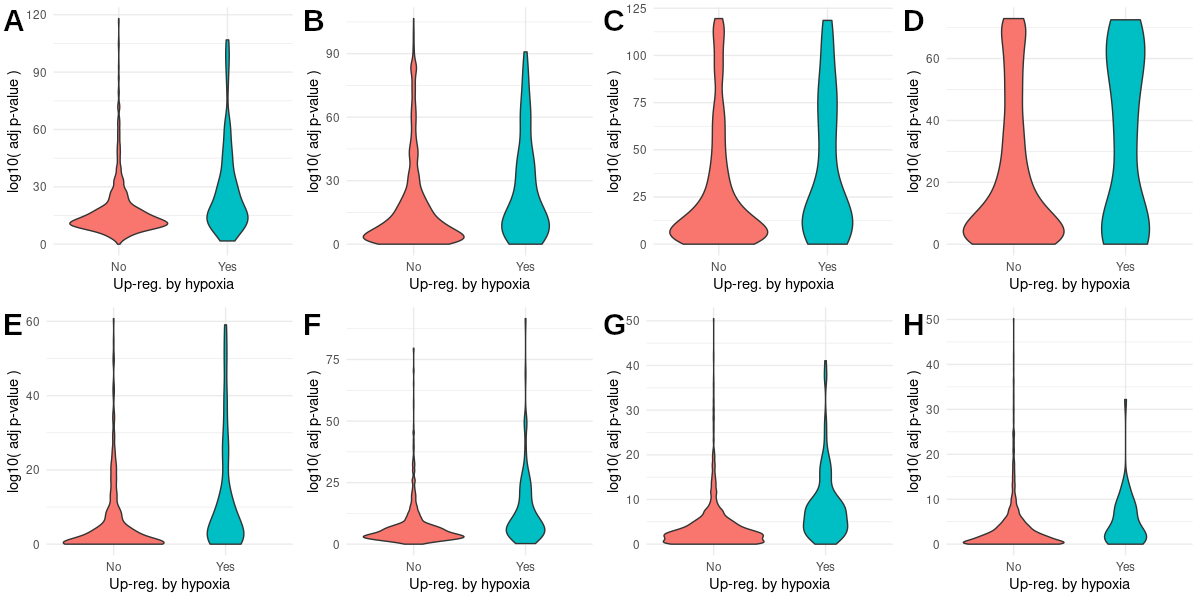

Supplement: Supplementary file 5 — Additional file 5: Fig. S2. Unsupervised clustering matches areas classified as hypoxic. UMAP representations (first column) and microscopy overlay (second column) of spot clustering using a shared nearest neighbor (SNN) based algorithm. Spots marked as hypoxic by the decision trees group together and ”hypoxic” clusters are positioned close by in the UMAP space. A: Human Prostate Cancer, Adenocarcinoma with Invasive Carcinoma (FFPE). B: Adult Mouse Kidney (FFPE). C: Human Glioblastoma. D: Human Colorectal Cancer. [file 12859_2022_4741_MOESM5_ESM.png]

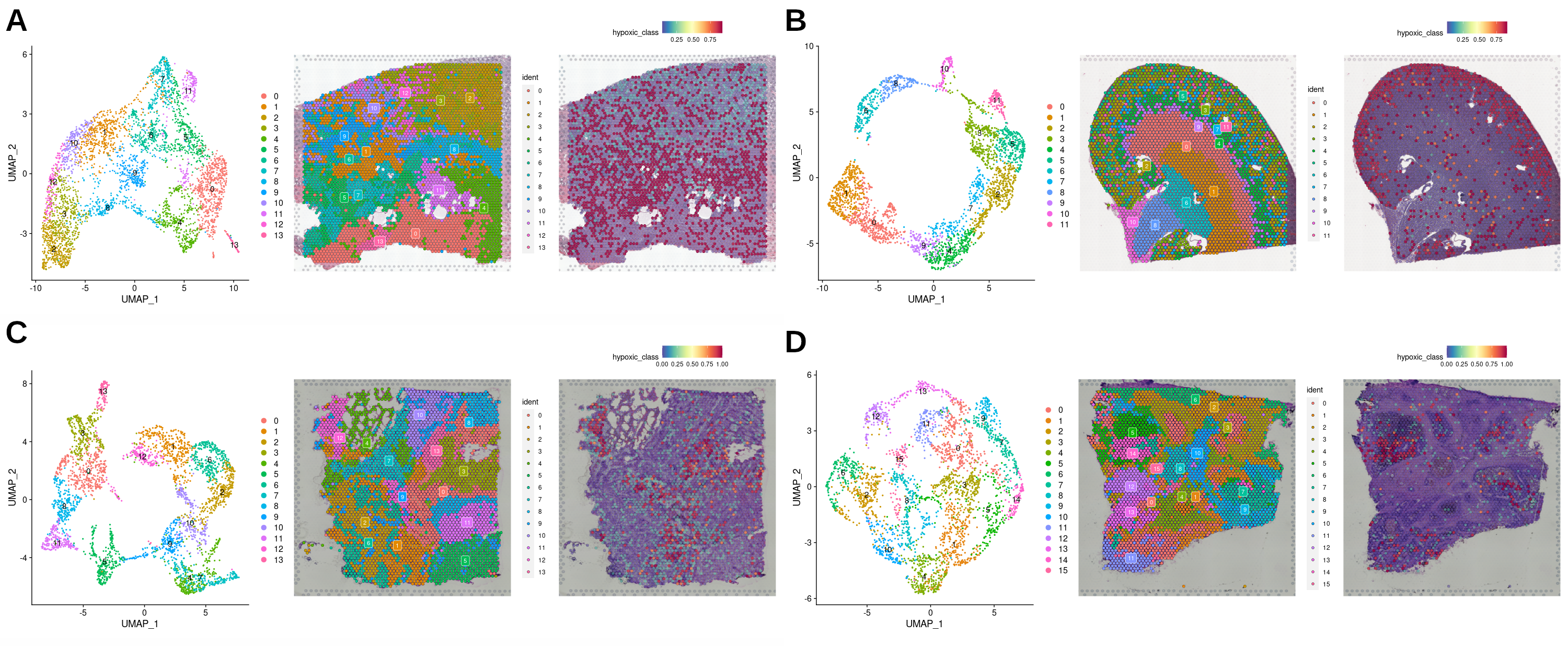

Supplement: Supplementary file 6 — Additional file 6: Fig. S3. Hypoxic genes DE between Visium dataset clusters. -Log10 (FDR adjusted p-values) of genes significantly up-regulated between clusters, according to their up-regulation status in the previously cited hypoxia meta-analysis. A: Prostate cancer, C7 vs C0. B: Prostate cancer, C11 vs C2. C: Mouse kidney, C3 vs C1. D: Mouse kidney, C5 vs C6.E: Glioblastoma, C12 vs C8.F: Glioblastoma, C1 vs C5 . G: Colorectal cancer, C14 vs C2. H: Colorectal cancer, C7 vs C8. [file 12859_2022_4741_MOESM6_ESM.png]
